# Effect of hyperglycemia on lung microbiota and treatment outcome in pulmonary tuberculosis: A scoping review

**DOI:** 10.12688/f1000research.159555.1

**Published:** 2024-12-20

**Authors:** Victor Moses Musyoki, Marianne Mureithi, Annamari Heikinheimo, Elizabeth Maleche-Obimbo, Dennis Kithinji, Susan Musau, Kariuki Njaanake, Omu Anzala

**Affiliations:** 1 Department of Medical Microbiology and Immunology, University of Nairobi, Nairobi, Nairobi County, 00202, Kenya; 2 KAVI-Institute of Clinical Research, University of Nairobi, Nairobi, Nairobi County, 00202, Kenya; 3 Tuberculosis and HIV co-infection Training Program in Kenya, Fogarty International Center, Bethesda, Maryland, 2220, USA; 4 Department of Veterinary Medicine, University of Helsinki, Helsinki, Uusimaa, 00014, Finland; 5 Department of Paediatrics and Child Health, University of Nairobi, Nairobi, Nairobi County, 00202, Kenya; 6 Department of Medical Laboratory Sciences, Meru University of Science and Technology, Meru, Meru County, 60200, Kenya; 7 Research Methodology, Medright Consulting LTD, Maua, Meru, 60600, Kenya

**Keywords:** Diabetes, Tuberculosis, Comorbidity, Lung microbiome, Immune response, Cytokines, Pathogenesis, Scoping review

## Abstract

The comorbidity due to pulmonary tuberculosis (TB) and diabetes mellitus (DM) is a global health problem, but its mechanism remains unclear. It is suspected that hyperglycemic alteration of the immune response to TB and the composition of the lung microbiota play an important role. This scoping review aimed to contribute to the understanding of the mechanisms by mapping evidence on the effect of hyperglycemia on physical health indicators, immune cell counts, cytokine levels, and the composition of lung microbiota in patients with the DM-TB comorbidity.

A systematic search for research articles about the relationship between hyperglycemia and physical health, immune cells, and cytokine levels in humans was conducted in MEDLINE, Scopus, and CINAHL Plus. Then, articles on the interactions between the immune cells, cytokines, and lung microbiota were identified through Google Scholar and Google search engines. Characteristics of the studies focusing on effects of hyperglycemia, the findings of the articles relevant to the research objectives, and strengths and weaknesses of the selected articles were charted in a data extraction tool.

Twenty-one articles on the effects of hyperglycemia on immune mediators and health outcomes of patients with DM-TB were included. The evidence showed hyperglycemia to be associated with unfavorable treatment outcomes; altered counts and functioning of dendritic cells, monocytes, and CD4+ T cells; and changes in cytokine levels (mainly INF-γ, IL-17, IL-1β, IL-2, IL-6, IL-10, and TNF-α) in patients with DM-TB. The composition of the lung microbiota changed in correlation with changes in physical health outcomes, counts of immune cells, and cytokine levels.

Thus, hyperglycemia, immune responses, and dysbiosis of the lung microbiota are integral in the pathogenesis of DM-TB and TB treatment outcomes. A prospective cohort study, especially in individuals with newly diagnosed DM versus known DM and concomitant latent TB versus active TB, is recommended to define causal relationships.

## Introduction

Pulmonary tuberculosis (PTB) is caused by *Mycobacterium tuberculosis* (MTB), which is a leading infectious agent cause of death globally ( Fukunaga *et al.*, 2021). PTB, more common than extra-pulmonary TB, affects about 80% of the patients with TB. Although the incidence of PTB has been declining over the years, it is still high with about 10 million people developing the disease in 2019 and approximately 1.4 million and 1.3 million people dying of TB in 2019 and 2022, respectively ( Fukunaga *et al.*, 2021; Menon *et al.*, 2020; World Health Organization, 2023).

The increasing prevalence of DM globally complicates the efforts to eradicate TB since DM-TB comorbidity makes patients non-responsive to conventional TB treatment regimen ( Menon *et al.*, 2020). Based on studies published between 1999 and 2017, the pooled prevalence of DM-TB in Sub-saharan Africa is 9% (95% CI: 6.0, 12.0%) ( Alebel *et al.*, 2019). The odds of multi-drug resistant TB (MDR-TB) in patients with DM can be twice the odds of MDR-TB in non-diabetic patients ( Ngo, Bartlett and Ronacher, 2021). In addition, patients with the DM-TB comorbidity are more MTB-infectious than patients with other comorbidities ( Ngo, Bartlett and Ronacher, 2021). Thus, the comorbidity derails efforts to reduce the incidence rate of TB and complicates the treatment of PTB based on the existing guidelines.

Infection with *M. tuberculosis* disturbs hormone and cytokine production to make the liver produce excess glucose and induce insulin resistance ( Bisht *et al.*, 2023; Rao *et al.*, 2019). MTB infection alters insulin activity, thus deranging carbohydrate metabolism, which impairs glucose tolerance and consequently increases blood glucose levels. Insulin resistance often occurs during active tuberculosis due to increase in pro-inflammatory cytokine levels and the dysregulation of lipid metabolism secondary to MTB infection ( Bisht *et al.*, 2023).

About 10% of patients with TB and who have embarked on TB treatment have hyperglycemia at 3 – 6 month follow-up, which could be predisposing the patients to complications and drug resistance ( Menon *et al.*, 2020). Half of the patients among whom hyperglycemia is detected at baseline have persistent hyperglycemia at 3 – 6 month follow-up. Patients with DM-TB comorbidity are more likely to have lower concentrations of antibiotics during treatment of MTB, which further complicates management ( Ngo, Bartlett and Ronacher, 2021). Therefore, hyperglycemia threatens the achievement of treatment outcomes in TB patients.

In euglycemic individuals, the lung microbiota play a significant role in health and disease including innate protection against pathogenic microorganisms. In patients with TB, hyperglycemia may contribute to dysbiosis of the lung microbiota, thus increasing the risk of disease progression ( Dumas *et al.*, 2018; Wang *et al.*, 2022). Lung microbiota is connected to the gut microbiota through the bidirectional gut-airway axis, which is a system for information exchange to modulate microorganism-immune interactions ( Özçam and Lynch, 2024). Wang et al. (2022) reported that the bacterial types and species diversity of the gut microbiota were low in a PTB group compared to a healthy group. The populations of the predominant beneficial bacteria in the intestinal microbiota, such as Firmicutes, were also significantly low ( Wang *et al.*, 2022; Comberiati *et al.*, 2021). Considering the gut-lung axis, hyperglycemia may also play a role in the disruption of the pulmonary microbial community ( Dumas *et al.*, 2018). Evidence on the effect of the hyperglycemia in causing lung dysbiosis and the microbiome-immune crosstalk among patients with TB is necessary to understand its implications in the pathophysiology of TB, effects of therapeutic interventions, and treatment outcomes ( Comberiati *et al.*, 2021).

The specific objectives of this scoping review include identifying the effects of hyperglycemia on cytokine levels, immune cells, and physical health of patients with TB, and exploring how the changes influence the lung microbiome to affect health outcomes in TB. The scoping review will clarify the implications of hyperglycemia in adult patients with MTB infection by synthesizing evidence on the immunological effects of the hyperglycemia on the lung microbiome. This evidence will be useful in elucidating the need to investigate the value of hyperglycemia screening and DM management in the care plan for patients with pulmonary TB.

## Methods

### Protocol design

This scoping review adhered to the The Preferred Reporting Items for Systematic Reviews and Meta-Analysis (PRISMA) extension for scoping reviews (PRISMA-ScR) checklist ( Kithinji *et al.*, 2024). A scoping review methodology was appropriate since evidence on the relationships between PTB, hyperglycemia, and the lung microbiome is scarce and published in research articles with heterogeneous methods. A review protocol does not exist.

### Literature search

***Eligibility criteria***

Peer-reviewed primary articles that explored the relationship between PTB, hyperglycemia, and lung dysbiosis were included in the study. The participants in the studies must have been aged over 18 years and diagnosed with PTB based on chest X-ray and bacteriological confirmation of the MTB infection. They must also have had hyperglycemia defined as fasting blood glucose >5.6 mmol/L, 2-hour oral glucose tolerance >7.8 mmol/L, or HbA1c >5.7%. The exclusion criteria eliminated studies whose outcomes were not objectively measured due to their susceptibility to bias. Studies published in non-English language and without English translations were also excluded because all the authors are English speakers.

***Information sources***

The MEDLINE, Scopus, and CINAHL Plus databases were searched to identify research studies that explored the relationship between MTB infection and hyperglycemia. The reference lists of the systematic reviews and meta-analyses retrieved from the search were screened to identify additional articles that would be included in this study. Google Scholar and Google were used to search and identify articles on the relationships between the significant changes in measured outcomes in the PTB - hyperglycemia publications with lung microbiota. Table 1 outlines the details of the searches conducted and the full search strategy for each source. The key search terms were tuberculosis, hyperglycemia, and lung microbiome. The key terms and their synonyms were combined using Boolean operators (AND, OR) to improve the sensitivity of the search ( Table 1).

**Table 1.** Search strategies for MEDLINE, Scopus, and CINAHL Plus databases.

Database	Date searched	Filters and limits	Search query	Hits
MEDLINE	13/06/2024	**Limiters** - Peer Reviewed; English Language; Human; Age Related: All Adult: 18+ years; Language: English **Expanders** - Apply equivalent subjects **Search modes** - Boolean/Phrase	AB (tuberculosis or tb) AND TX (hyperglycemia or high blood sugar or high blood glucose)	71
Scopus	14/06/2024	**Refine search** **Subject area:** immunology and microbiology **Document type**: article **Language:** English **Source type:** journal	(tuberculosis AND hyperglycemia) AND ( LIMIT-TO (SRCTYPE, "j")) AND ( LIMIT-TO (DOCTYPE, "ar")) AND ( LIMIT-TO (LANGUAGE, "English")) AND (LIMIT-TO (EXACTKEYWORD, "Human") OR LIMIT-TO (EXACTKEYWORD, "Humans"))	381
CINAHL Plus	14/06/2024	**Expanders:** Apply related words, apply equivalent subjects **Source types:** academic journals **Language:** English	TI (tuberculosis or tb) AND AB hyperglycemia	31

***Selection of sources of evidence***

Titles and abstracts of the identified articles were screened to identify articles that met the inclusion and exclusion criteria. The full-texts of the relevant records were read to assess suitability for the study objectives. The study population, outcome measures, and results of the data analysis were examined to only select articles that explored the changes in physical health, immune cells, and cytokine levels in the interplay between pulmonary TB and hyperglycemia. Two reviewers, VMM and DK, a medical microbiologist and a research methodologist, respectively, scrutinized each article to identify relevant data regarding the effect of hyperglycemia on physical health, immune cell counts, and cytokine levels in patients with PTB. The remarkable changes in outcomes observed in the included TB-hyperglycemia studies informed the search for articles on the relationship between the changes and dysbiosis in the lung microbiota.

### Data charting process

The researchers charted the data using a Microsoft excel data abstraction tool that captured information on the sample characteristics and the variables on the interaction between pulmonary TB and hyperglycemia. The two reviewers, VMM and DK, discussed any disagreements in their charting, which they resolved by engaging the co-authors to arrive at a majority decision.

***Data items***

The main data items included hyperglycemia in both pre-DM and DM, and PTB diagnosed through imaging or bacteriology as independent variables. The other data items included indicators of physical health in PTB and changes in immune cell counts and cytokine levels attributable to hyperglycemia. Changes in lung microbiota associated with changes in physical health, immune cell counts, and cytokine levels were the relevant data items in the second round of the search process. Details of the data items are provided in Table 2.

**Table 2.** Data items extracted from the selected articles.

Variable	Definition	Assumption
Hyperglycemia	Fasting blood glucose (FBG) >5.6mmol/L, impaired glucose tolerance – 2-hour Oral glucose tolerance test result of >7.8mmo/L or HbA1c levels of 5.7% – 6.4%	Hyperglycemia could be due to DM or other causes such as stress or TB-induced
Diabetes mellitus (DM)	A chronic condition characterized by hyperglycemia (FBS> 7 mmol/L, random blood sugar (RBS) or 2-hr postprandial glucose levels of 11 mmol/L, or HbA1c > 6.5%) due to impaired glucose metabolism	DM contributed to the hyperglycemia that affected the prognosis of TB
Prediabetes	A precursor of DM characterized by hyperglycemia (FBG = 5.5 – 6.9 mmol/L, 2-hour postprandial glucose 7.8-11 mmol/l, or HbA1c of 5.7% and 6.4%)	Even hyperglycemia that is yet to be DM can influence the pathogenesis of TB
Pulmonary tuberculosis (TB)	A lung infection caused by *Mycobacterium tuberculosis* diagnosed via chest X-ray and/or bacteriological sputum analysis (microscopy or culture)	Newly diagnosed TB, relapse and recurrent TB differ in prognosis and pathogenesis
PreDM or DM-TB comorbidity	The coexistence of hyperglycemia and TB diagnosis	The hyperglycemia worsened the course of TB
Cytokines	Signaling molecules that facilitate communication between cells particularly during inflammation	Hyperglycemia influences changes in cytokine levels, which disrupt lung microbiota to worsen the course of TB.
Lung microbiota	The pulmonary microbial community in the lower respiratory tract.	Hyperglycemia disrupts lung microbiota to cause poor health outcomes in patients with TB

### Synthesis of results

VMM and DK synthesized evidence on the effect of hyperglycemia on TB prognosis by grouping related evidence. The effects of hyperglycemia on physical indicators of health such as TB treatment failure and cavitation in the lungs upon chest X-ray; immune cells; and cytokines extracted from each of the sources were synthesized to elucidate the mechanism. The main strengths and weaknesses of the individual studies were considered during the synthesis to reduce the risk of bias.

## Results

### Selection of sources of evidence

**Figure 1.**
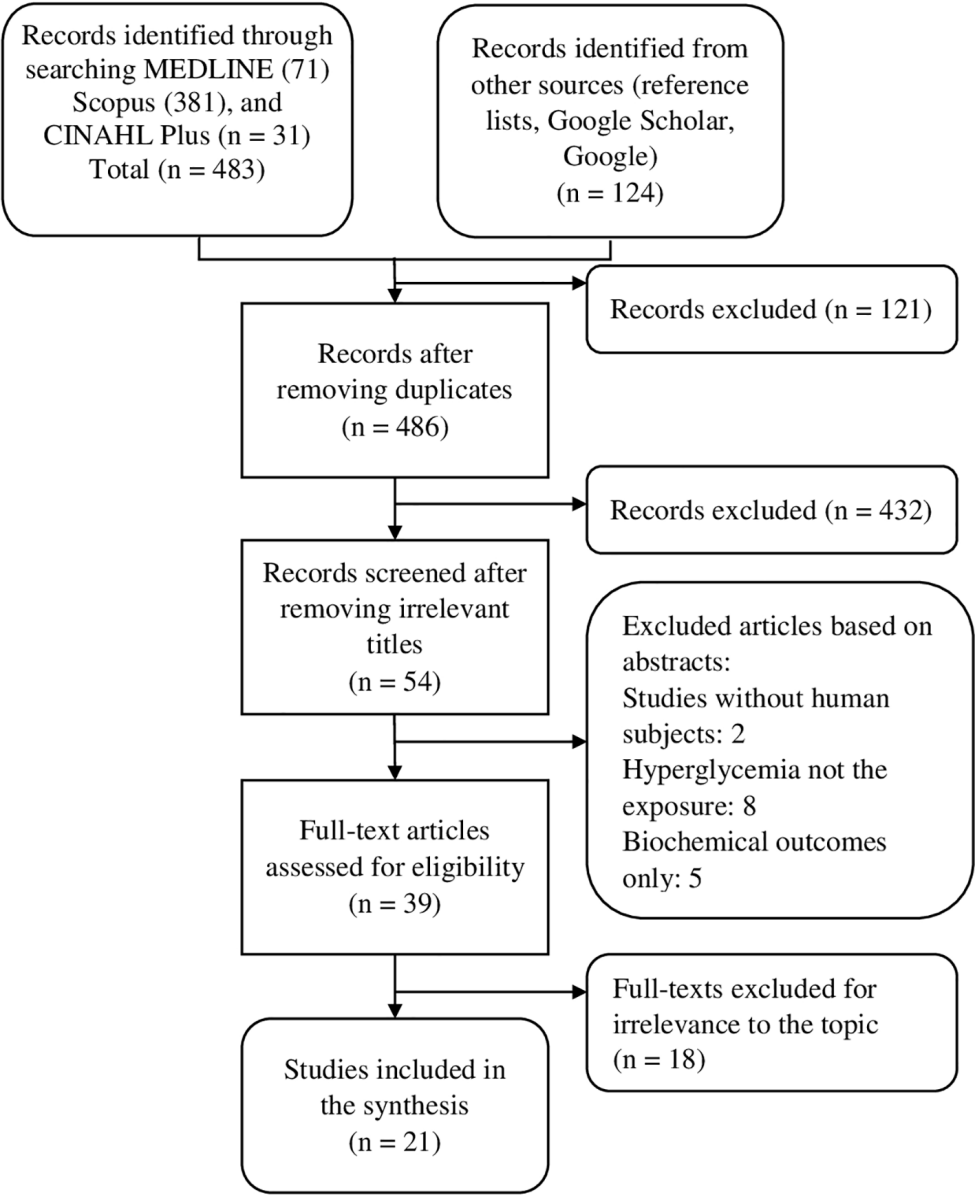
PRISMA flow diagram showing the selection of sources of evidence for the relationship between TB and hyperglycemia.

A total of 31 primary research articles were synthesized in this scoping review. Twenty-one were on the effects of hyperglycemia on various indicators of health in TB while 10 were on the effect of the changes in the indicators on the lung microbiota. Figure 1 shows the sources of evidence selected, screened, assessed for eligibility, and included in the review regarding the relationship between TB and hyperglycemia.

### Characteristics and results of individual sources of evidence

Table 3 (Extended data; Kithinji *et al.*, 2024) summarizes the details of the primary articles that were included in this scoping review. In instances where the study design was not identified in the article, it was deciphered from the description of the recruitment of participants and data collection processes.

### Synthesis of results

***Effects of hyperglycemia on overall TB treatment outcomes***

The results on the effects of hyperglycemia on overall TB treatment outcomes are shown in Table 4 (Extended data; Kithinji *et al.*, 2024). The findings show that hyperglycemia contributed to poor treatment outcomes among patients with TB ( Lin *et al.*, 2020; Liu *et al.*, 2021; Meng *et al.*, 2023; Pardeshi *et al.* 2024; Faurholt-Jepsen *et al., *2013). A prospective cohort study in six clinics in Jilin province, China reported a statistically significant association between persistent hyperglycemia of any duration and unfavorable treatment outcomes among patients with TB ( Lin *et al.*, 2020). However, the study relied on clinical presentation of patients to classify them as TB-negative, thus giving rise to a risk of overdiagnosis. Pardeshi *et al.* (2024) and Liu *et al.* (2021) also found that patients with TB and transient hyperglycemia had 2-4 times the odds of treatment failure compared to euglycemic patients. Although the estimates of the incidence rates of events were imprecise based on the wide confidence intervals, they were significant and showed a temporal relationship between hyperglycemia and poor TB treatment outcomes. Similarly, Faurholt-Jepsen *et al.* (2013) reported that the risk of mortality among patients with DM-TB in Mwanza, Tanzania was five times the risk of mortality among patients with TB only within the first 100 days after initiation of TB treatment. Considering the large sample size and the cohort study design, the evidence presented by Faurholt-Jepsen *et al.* (2013) cements the relationship observed between hyperglycemia and poor treatment outcomes in the various studies.

Poor treatment outcomes co-occured with dysbiosis of the lung microbiota ( Ticlla *et al.*, 2021; Wu *et al.*, 2013). Wu *et al.* (2013) found that dysbiosis in the lung microbiota is associated with poor TB treatment outcomes, treatment failure, and recurrence. Although Wu *et al.* (2013) analyzed sputum rather than bronchoalveolar lavage to characterize the lung microbiota, their findings suggest dysbiosis as a significant determinant of TB treatment outcomes. Ticlla *et al.* (2021), who sought to identify the bacteria that constitute lung microbiota in TB by analyzing the bronchoalveolar lavage fluid obtained from both lungs of patients with unilateral TB lesions, found that *Cupriavidus* spp *.* rather than *Streptococcus* spp. was the most dominant bacterial genus in the lower respiratory tract of patients with TB.

The form of dysbiosis in the lung microbiota is dependent on the pulmonary TB status. Wu *et al.* (2013) compared sputum microbiota across patients with three types of TB: new, recurrent and treatment-failure TB among 25, 30, and 20 patients, respectively. Higher abundance of *Bulleidia* spp *.* and *Atopobium* spp. was observed in patients with recurrent TB compared to newly diagnosed patients. The *Pseudomonas* / *Mycobacterium* and *Treponema* / *Mycobacterium* ratios were higher and lower, respectively, in patients with recurrent TB than in newly diagnosed TB patients. Similarly, in treatment failure TB, the *Pseudomonas* / *Mycobacterium* ratio was higher than in newly diagnosed cases ( Wu *et al.*, 2013). Patients with recurrent TB had more abundant *Corynebacterium* spp. compared to patients with treatment failure TB. On the other hand, the more abundant genera in the patients with treatment-failure TB compared to patients with recurrent TB included *Campylobacter, Prevotella, Atopobium, Blastobacter,* and *Treponema* ( Wu *et al.*, 2013).

Studies on lung microbiome have reported different compositions of the microbiota concurrent with variations in TB treatment outcomes; hence the TB’s pathogenesis could be due to the interplay of both hyperglycemia and lung dysbiosis. Species in the phylum firmicutes including *Staphylococcus* and *Selenomonas* were more abundant in the lung microbiota of patients with TB compared to patients without TB ( Ding *et al.*, 2021; Ticlla *et al.*, 2021). Abundance of members of phylum Actinobacteria such as *Mycobacteria* was also evident in the lung microbiota of patients with TB compared to those without it ( Ticlla *et al.*, 2021). *Porphyromonas* in the phylum Bacteroidetes and *Fusobacterium* in the phylum Fusobacteria were more common compared to *Streptococcus* in the phylum Firmicutes in the sputum of patients with TB ( Ding *et al.*, 2021).

Poor physical health outcomes in TB were more common in hyperglycemic patients than in euglycemic patients ( Barreda *et al., *2020; Meng *et al.*, 2023; Meng *et al.*, 2022). Both the retrospective cross-sectional study by Barreda *et al.* (2020) and the cohort study by Meng *et al.* (2023) reported that hyperglycemic patients with TB were more likely to have various types of pulmonary lesions such as cavities, alveolar infiltrates, and fibrous tracts compared to euglycemic patients. The two-year follow-up period in the study by Meng *et al.* (2023) allowed the identification of hyperglycemia as a risk factor of poor health outcomes. The poorer the glycemic control, the higher the rates of pulmonary lesions. Byashalira *et al.* (2022) improved health outcomes of patients with DM-TB by integrating glycemic control into a TB and HIV care programme.

***Effects of hyperglycemia on TB immunity***

Results on the effects of hyperglycemia on the immunological markers of health in TB (Table 5 (Extended data; Kithinji *et al.*, 2024)) show that hyperglycemia reduced the population and functioning of dendritic cells and macrophages in patients with DM-TB ( Gomez *et al.*, 2013; Kumar *et al.*, 2017). Kumar *et al.* (2017) observed a negative correlation between hyperglycemia and the frequencies of monocytes, plasmacytoid, and myeloid dendritic cells, as well as intermediate and classical monocytes, upon phenotyping monocytes and dendritic cells *ex vivo* from patients with DM-TB comorbidity and others with only TB under standard TB treatment. Although the correlations were borderline, perhaps due to the small sample size and the cross-sectional study design, the results pointed to hyperglycemia’s downregulation of the functioning of monocytes and dendritic cells in patients with TB ( Kumar *et al.*, 2017). Another *ex vivo* study revealed lower attachment and phagocytosis of MTB in monocytes from patients with DM, especially upon heat inactivation of serum in the case of poorly controlled hyperglycemia ( Gomez *et al.*, 2013). This suggests that hyperglycemia could impair the functioning of monocytes against MTB by inhibiting their activation through the complement system. Similarly, multiple *ex-vivo* experiments showed that patients with DM-TB have significantly low complement receptor 3 or CD11b and MARCO levels compared to patients with TB, which translated to low capacity for phagocytosis in macrophages ( Panda *et al.*, 2022).

Hyperglycemia variedly affected the populations of different types of T-cells. A prospective comparative study demonstrated that within two months of TB treatment and follow-up, patients with DM-TB had lower CD4+ T cell counts compared to patients with TB only, DM only, and healthy controls ( Rendón Ramírez *et al.*, 2023). Although the study could have been underpowered and ungeneralizable due to a small sample recruited from a single hospital, its findings indicate the possibility of DM contributing to a decrease in CD4+ T cells count. On the other hand, a prospective observational cohort study reported that increased HbA1c in the course of TB treatment is associated with elevated Th1 and Th17 CD4+ responses ( Krause *et al.*, 2023). In an experimental study, the numbers of Th17 CD4+ T cells and a regulatory T cell subset, RORγt ^+^ Tregs, changed depending on the presence of *Lactobacillus murinus* ( *Lagilactobacillus murinus*) strain (CNCM I-5314), which is a pulmonary Lactobacillus strain ( Bernard-Raichon *et al.*, 2021). Intranasal introduction of the CNCM I-5314 and not intragastrically in mice infected with MTB increased the expansion of lung leukocytes, particularly Th17 CD4+ T cells and RORγt ^+^ Tregs locally in the lungs and airways ( Bernard-Raichon *et al.*, 2021). Upon administering the CNCM I-5314, the proportion of RORγt ^+^ Tregs increased most prominently since they changed from 14% to 50% of all the lung Foxp3 ^+^ CD41 T cells in MTB-infected mice.

In an experiment that tested the activation of immune cells from patients with active and latent TB by stimulating the peripheral blood mononuclear cells with *Mycobacterium bovis* BCG, an inverse correlation was observed between the level of glycemia and IFN-γ- and TNF-α- CD4+ T cell productions ( Boillat-Blanco *et al.*, 2018). This suggested that hyperglycemia could be downregulating antigen processing and presentation of the whole MTB, thus stalling the production of cytokines that promote TB immunity. Although the study did not include patients with transient hyperglycemia, the findings among patients with DM empirically suggest the effect of hyperglycemia on TB immunity.

Findings from different studies show that hyperglycemia could be disrupting cytokine responses in favor of TB immunopathology. An *ex-vivo* experimental study on the mRNA expression of pro-inflammatory and anti-inflammatory cytokines in monocyte-derived macrophages reported a positive correlation between IL-1β expression and HbA1c levels ( Panda *et al.*, 2024). Levels of IL-1β were also elevated among patients with DM-TB compared to patients with DM only or TB only ( Kumar *et al.*, 2020). In contrast, lower levels of IL-1β were reported in patients with DM- TB comorbidity compared to patients with TB only ( Chao *et al.*, 2015).

IFN-γ levels were low in newly diagnosed DM and TB comorbidity cases. Patients with newly diagnosed DM (NDM) were characterized by significantly diminished unstimulated levels of INF-γ ( Kumar *et al.*, 2020; Panda *et al.*, 2024). Additionally, INF-γ levels were positively correlated with IL-β levels ( Panda *et al.*, 2024). Earlier, Kumar *et al*. (2014) reported that latent TB in treatment-naïve patients with DM or pre-DM was associated with diminished circulating levels of INF-γ.

IFN-γ levels were elevated in patients with known DM-TB comorbidity compared to patients with DM only ( Chen *et al.*, 2022; Kumar *et al.*, 2020). Although the power of the study by Chen *et al.* (2022) was low due to a small sample size, the observed differences between the compared age- and sex-matched groups suggest a possible effect of the hyperglycemia on the cytokine levels. Similar findings have been reported from a cross-sectional study indicating that INF-γ levels were more raised in patients with DM but with controlled hyperglycemia and latent TB than in healthy endemic controls upon purified protein derivative antigen (PPD) stimulation ( Masood *et al.*, 2022).

Alteration of the lung microbiota affected the production of INF-γ ( Bernard-Raichon *et al.*, 2021; Palma Albornoz *et al.*, 2021; Xia *et al.*, 2022). The introduction of CNCM I-5314 into the lung microbiota significantly decreased the production of INF-γ ( Bernard-Raichon *et al.*, 2021). Dysbiosis characterized by increased *Bacteroides* and *Firmicutes* in the lungs decreased the number of IFN-γ-producing CD4+ cells and consequently IFN-γ levels ( Palma Albornoz *et al.*, 2021). Xia *et al.* (2022) reported a positive correlation between IFN-γ and *Leptotrichia* and *Gemella,* which are members of the lung microbiota in healthy control populations.

IL-10 levels in DM-TB are inconsistent across various studies. For instance, IL-10 levels were significantly low in patients with increased HbA1c ( Krause *et al.*, 2023). In contrast, findings from a study by Chen and colleagues showed that the levels in DM-TB patients were double those in patients with DM only or TB only ( Chen *et al.*, 2022). In another *in vitro* study, IL-10 levels were significantly enhanced in the mice infected with the MS1987 protein (the virulence protein for MTB) especially after intranasal infection with anaerobic *Escherichia coli* and *Delftia acidovorans* ( Fraser-pitt *et al.*, 2023).

Significantly high IL-6 levels were reported among patients with DM-TB compared to patients with TB only or DM only, even in cases with latent TB ( Chen *et al.*, 2022; Kumar *et al.*, 2020; Masood *et al.*, 2022). This upregulation was mainly in patients with HbA1c levels greater than 8 g/dL. Contrary to these findings, an *in vitro* study reported a significant decrease in IL-6 in mice at day 9 post-infection with a model containing the MS1987 protein ( Fraser-pitt *et al.*, 2023). Intranassally introducing *E. coli* into mice increased the production of IL-6 while exposure to *D. acidovorans* inhibited IL-6 production. Xia *et al.* (2022) also reported a negative correlation between IL-6 levels and *Corynebacterium* in patients with TB.

IL-17 levels were elevated among DM-TB patients ( Chen *et al.*, 2022). Similarly, IL-17A levels were high in patients with DM-TB but diminished in patients with newly diagnosed DM and TB comorbidity ( Kumar *et al.*, 2020). Kumar *et al.* (2014) also reported diminished levels of IL-17F in patients with latent TB and DM or pre-DM. Fraser-pitt *et al.* (2023) found that models of MTB with the pathogenic protein MS1987 caused a decrease in levels of 1L-17 in the lungs at day nine post-infection. Administering *E. coli* intranassaly into mice led to increased production of IL-17. Administering CNCM-I-5314 caused the lung CD4+ T cells to produce more IL-17 and express more IL-17a mRNA ( Bernard-Raichon *et al.*, 2021). Xia *et al.* (2022) reported that IL-17 was positively correlated with *Gemella* and *Streptococcus* of the lung microbiota.

TNF-α levels varied depending on the status of the DM and TB. They were elevated in DM-latent TB cases upon PPD stimulation ( Masood *et al.*, 2022). Kumar *et al.* (2020) also reported increased levels of TNF-α in patients with DM-TB but the unstimulated levels were diminished in patients with newly diagnosed DM and TB comorbidity. Similarly, the circulating levels of TNF-α were diminished in patients with DM and pre-DM, and latent TB ( Masood *et al.*, 2022; Chen *et al.*, 2022). Bernard-Raichon *et al.* (2021) reported significantly decreased production of TNF-α in lung exudates upon CMCM I-5314 treatment. Intranasal administration of *D. acidovorans* was shown to inhibit TNF-α production ( Fraser-pitt *et al.*, 2023).

IL-2 levels also varied depending on the status of the DM-TB. They were elevated in DM-latent TB cases compared to healthy endemic controls upon PPD-stimulation ( Masood *et al.*, 2022; Kumar *et al.*, 2020). Unstimulated levels were significantly low in patients with newly diagnosed DM and TB comorbidity ( Kumar *et al.*, 2020). Similarly, diminished circulating levels of IL-2 were associated with latent TB infection in DM or pre-DM patients ( Kumar *et al.*, 2014).

## Discussion

This scoping review sought to identify the effects of hyperglycemia on cytokine levels, immune cells, and physical health of patients with TB and explore how the changes influence the lung microbiome to affect health outcomes in TB.

Overall, we found that hyperglycemia is associated with poor health outcomes, and changes in cytokine levels, altered counts and functioning of dendritic cells, monocytes, and CD4+ T cells in patients with DM-TB. The microbial lung composition changes in correlation with changes in physical health outcomes, counts of immune cells, and cytokine levels. Cavities, fibrous tracts, and alveolar infiltrates among other pulmonary lesions commonly occur in patients with DM-TB. These patients experience treatment failure, recurrence, and death ( Kornfeld *et al.*, 2020).

### Effects of hyperglycemia on immune responses of patients with DM-TB

Dendritic cells and monocytes are central in the presentation of MTB antigens to the naïve T-cells in order to activate the adaptive immune response but their numbers are decreased in DM ( Comberiati *et al.*, 2021). T-cell-mediated adaptive immune responses are integral in controlling MTB infections in humans ( Jasenosky *et al.*, 2015). For instance, the availability of polyfunctional T cells, which are desirable in the fight against TB due to their capacity for effector and proliferative abilities considering their trifunctional production of TNF, IFN-γ, and IL-2 determines TB progression ( Jasenosky *et al.*, 2015). Therefore, a decrease in CD4+ T cells during DM-TB comorbidity may impede the induction and maintenance of the adaptive immunity against TB.

The contrasting findings on IL-1β levels in similar studies among patients with DM-TB suggests that the production of IL-1β may be influenced by other factors such as the status of DM or TB. MTB decreases the activation of pyrin and AIM2- domain containing 3 (NLRP3)-inflammasomes, which induces the production of INF-β that subsequently reduces the secretion of IL-1β ( Etna *et al.*, 2014). A prospective cohort study that follows up patients with TB and hyperglycemia in the course of disease and treatment is essential to define the factors behind varying patterns of IL-1β levels among patients with DM-TB.

IFN-γ levels are low in newly diagnosed DM-TB and high in known DM-TB cases. When diabetes status is known, treatment is often initiated, hence hyperglycemia is controlled ( Li *et al.*, 2020). It is therefore important to control hyperglycemia in DM-TB to reduce the blunting of IFN-γ production. However, this is a double edged sword as the reduction of the pro-inflammatory IFN-γ in hyperglycemia not only slows down the inflammation-based pathology of TB but also hinders killing of the bacteria, induction of nitric oxide synthesis, and macrophage activation that depend on IFN-γ ( Etna *et al.*, 2014).

In newly diagnosed DM-TB, IL-2, IL-17, and TNF-α levels are low, while in known DM-TB, IL-2, IL-17, and TNF-α levels are high. Chronic hyperglycemia rather than acute hyperglycemia is important in inducing inflammation in the lungs through cytokines such as IL-2, IL-17, and TNF-α ( Ngo, Bartlett and Ronacher, 2021). IL-17 is involved in the immune defense against bacteria, including MTB. As a pluripotent pro-inflammatory cytokine, it recruits neutrophils, induces chemokines’ and cytokines’ production, modifies the differentiation of T-helper cells, and prompts the release of antimicrobial proteins that mediate inflammation in TB ( Chung, Ye and Iwakura, 2021). Similarly, TNF-α, which induces bactericidal effects in macrophages and has pro-inflammatory effects ( Yuk *et al.*, 2024), is low in newly diagnosed DM-TB but high in known DM-TB. Its levels are also high in active TB ( Suzukawa *et al.*, 2020), which may explain why they are elevated in DM-TB but suppressed in newly diagnosed DM and TB comorbidity. IL-2 is central in adaptive immune responses and immune tolerance ( Damoiseaux, 2020), hence alterations of its levels by hyperglycemia may have adverse health implications.

### Effects of hyperglycemia-instigated changes on the lung microbiome in TB

The lungs are not sterile since bacteria and fungi dominate the lower airways. *Mycobacteria* spp. are present in the lower airways of both individuals with and without TB, although they are dominant among patients with TB ( Hu *et al.*, 2020). This scoping review demonstrates that patients with TB have different compositions of lung microbiota with varying patterns. *Pseudomonas*, *Acinetobacter, Porphyromonas, Bulleidia,* and *Selemonas* were abundant while *Treponema, Streptococcus,* and *Prevotella* were scanty in patients with TB compared to healthy controls. Other studies have reported the abundance of *Gramulicatella* and *Pseudomonas* in the lung microbiota of patients with TB ( Wu *et al.*, 2013). *Sharpea* and *Bergeyella* were distinctively detected in the sputum of patients with TB. The dominant genera in healthy subjects include *Streptococcus, Selenomonas, Prevotella, Bifidobacterium,* and *Neisseria.* Others include *Leptotrichia, Catonella, Treponema,* and *Coprococcus* ( Wu *et al.*, 2013). The fungal members of the lung microbiome belong to the phyla Ascomycota and Basidiomycota ( Hu *et al.*, 2020). Patients with treatment failure TB had more abundant *Pseudomonas* than patients with newly diagnosed TB while *Corynebacterium* was more common in recurrent TB compared to treatment failure TB ( Wu *et al.*, 2013). Therefore, there is varying lung dysbiosis in patients with different TB status, which implies that patients with DM-TB comorbidity may have a distinct composition of lung microbiome.

The lung microbiome remains vital in determining pulmonary health and disease. The poor treatment outcomes evident in patients with both TB and hyperglycemia indicate the possibility of hyperglycemia contributing to the lung dysbiosis. Since studies on lung microbiome have also reported variations in composition of the microbiota based on TB treatment outcomes, TB pathogenesis could be due to the interplay of both hyperglycemia and lung microbial dysbiosis. Hyperglycemia is commonly associated with dysbiosis in the gut ( Darra *et al.*, 2023), it is therefore likely to be associated with dysbiosis in the lungs given the gut-lung axis. Thus, there is a need to determine the relationship between hyperglycemia and dysbiosis in the lung microbiota among patients with TB.

Changes in T-cells populations such as regulatory T cells, which the composition of lung microbiota licenses, are central in the control of pathogenesis of TB ( Mori, Morrison and Blumenthal, 2021). As a result, in patients with DM-TB, hyperglycemia could influence lung dysbiosis, subsequently reducing the production of polyfunctional T cells and compromising the adaptive immune response against TB.

The present review of literature did not find evidence on the interactions between lung microbiota and dendritic cells in patients with TB. However, some members of the microbiota may interact with the dendritic cells to produce cytokines that promote type 1 immunity ( Koizumi *et al.*, 2008). Similarly, the interaction between commensal bacteria through their metabolites and the immune system can contribute to the differentiation of monocytes to macrophages and boost the host innate defense against MTB ( Schulthess *et al.*, 2019). Thus, dendritic cells and monocytes may mediate the interactions between the lung microbiome and hyperglycemia, thus influencing the course of TB in patients with DM-TB comorbidity.

Changes in the levels of IFN-γ, IL-17, and TNF-α, which are associated with both hyperglycemia and lung dysbiosis, are key players in the inflammatory circuits that control the progression of TB ( Mori, Morrison and Blumenthal, 2021). For instance, less IFN-γ is produced in TB disease states when Bacteroidetes and Firmicutes are dominant in the lung microbiome. On the other hand, more IFN-γ is produced in health state when *Gemella* and *Leptotrichia* dominate the lung microbiota in healthy individuals. IL-10, which is produced to modulate the pathogenesis of TB, acts by dampening excess inflammation ( Etna *et al.*, 2014). The increase in IL-10 levels with the intranasal administration of *E. coli* and *D. acidovorans* implies that lung dysbiosis contributes to IL-10 levels variation in enhancing or dampening inflammation. Similarly, lung dysbiosis affects IL-17 levels as evident in upsurge in IL-17 when dysbiosis was altered via intranasal exposure to *E. coli, Gemella, Lactobacillus,* and *Streptococcus.* This scoping review also shows that TNF-α levels were diminished upon intranasal introduction of *Lactobacillus* and *D. acidovans* in the lungs. Thus, hyperglycemia may contribute to the pathogenesis of TB by altering the lung microbiota, which then disorients the immune response to TB through changes in cytokine levels.

Increase in IL-6 levels, common in patients with DM-TB, could be due to alterations in the composition of the lung microbiome. In this scoping review, we found that, IL-6 levels in mice infected with TB could be increased by intranasally introducing *E. coli* or reduced by administering *D. acidovorans.* *Corynebacterium*, whose low population in the lung microbiota is associated with increased IL-6 levels, is a major immunomodulatory bacterium in the lung microbiota ( Liu *et al.*, 2022). IL-6 is in the pro-inflammatory cytokine family and a major player in the human cytokine network ( Uciechowski and Dempke, 2020), hence variations in its levels should be explored when studying the relationship between hyperglycemia and lung microbiota in TB pathogenesis.

The main weakness of this scoping review is that majority of the included studies analyzed sputum samples to assess lung microbiome. There was, therefore, a possibility of specimen contamination with bacterial genera that are common in the oropharynx such as *Prevotella, Atopobium,* and *Bulleidia.* As a result, it is likely that the oropharynx microbiota would be misclassified for lung microbiota ( Mori, Morrison and Blumenthal, 2021; Goddard *et al.*, 2012). This underlines the need to consider bronchoalveolar lavage (BAL) specimen for an accurate depiction of the lower respiratory system microbiota.

## Conclusion

In conclusion, hyperglycemia and lung microbiota are involved in the pathogenesis of DM-TB, which culminates in poor treatment outcomes. They mediate their effects through changes in counts of dendritic cells, monocytes, CD4+ T cells, and cytokine levels. The interactions between hyperglycemia and the immune cells, cytokines, and lung microbiota vary depending on whether the DM is newly diagnosed or its a known case and whether TB is latent or active. A prospective study is necessary to determine the causal relationships in the interactions between hyperglycemia, immune cells, cytokines, and lung microbiota that result in poor TB treatment outcomes.

### Ethics and consent

Ethical approval and consent were not required.

## Author contributions

**Conceptualization:** Victor MM, Dennis K, Kariuki N, Elizabeth MO, Omu A

**Data curation, formal analysis, investigation:** Victor MM, Dennis K

**Methodology:** Victor MM, Dennis K, Susan M, Kariuki N, Elizabeth MO

**Supervision, validation:** Elizabeth MO, Kariuki N, Omu A, Marianne M, Annamari H

**Writing – original draft preparation:** Victor MM, Dennis K

**Writing - review and editing:** Victor MM, Dennis K, Elizabeth MO, Susan M, Kariuki N

## Data Availability

No data are associated with this article. Open Science Framework: Materials and data for the DM-TB immunopathology and lung dysbiosis scoping review. https://doi.org/10.17605/OSF.IO/WM82P ( Kithinji *et al.*, 2024). This project contains the following extended data:
• The filled PRISMA-ScR checklist. •
Table 3 showing characteristics of the selected studies. •
Table 4 with data on physical health outcomes in patients with TB extracted from selected sources. •
Table 5 with data on immunological markers of health in patients with TB extracted from the included articles. Data are available under a Creative Commons (CC0 1.0 Universal) license.
