# Supplementary material for: Effect of hyperglycemia on lung microbiota and treatment outcome in pulmonary tuberculosis: A scoping review
Source: F1000Res. 2024 Dec 20;13:1543. [Version 1] doi: 10.12688/f1000research.159555.1 (PMC11840297; doi:10.12688/f1000research.159555.1)
Supplement: Peer review history

# Reviewer report: Reviewer response for version 1

**DOI:** 10.5256/f1000research.175303.r362451

**Published:** 2025-02-19

**Authors:** Sonal Sekhar Miraj

**Affiliations:** 1 Manipal Academy of High Education, Manipal, India

1. In the abstract, the authors mention, "The composition of the lung microbiota changed in correlation with changes in physical health outcomes, counts of immune cells, and cytokine levels." What way composition of microbiota should be mention (phylum levels, genes level or microbial diversity)

 2. Effect of the hyperglycemia on lung microbiota, the readers will expect changes in the microbial diversity and richness. These points are not discussed in the manuscript

 3.  How can you ensure the dysbiosis in the lung microbiota is due to hyperglycemia alone, because TB and its ATT drugs itself can cause the dysbiosis.

 4. In conclusion, authors mention "hyperglycemia and lung microbiota are involved in the pathogenesis of DM-TB, .........."

 Is it pathogenesis of DM-TB? this is not clear

Are the rationale for, and objectives of, the Systematic Review clearly stated?

Yes

Is the statistical analysis and its interpretation appropriate?

Not applicable

If this is a Living Systematic Review, is the ‘living’ method appropriate and is the search schedule clearly defined and justified? (‘Living Systematic Review’ or a variation of this term should be included in the title.)

Not applicable

Are sufficient details of the methods and analysis provided to allow replication by others?

Yes

Are the conclusions drawn adequately supported by the results presented in the review?

Yes

Reviewer Expertise:

Diabetes and Tuberculosis

I confirm that I have read this submission and believe that I have an appropriate level of expertise to confirm that it is of an acceptable scientific standard, however I have significant reservations, as outlined above.

# Reviewer report: Reviewer response for version 1

**DOI:** 10.5256/f1000research.175303.r362457

**Published:** 2025-02-12

**Authors:** Tahziba Hussain

**Affiliations:** 1 HIV/AIDS UNIT, ICMR-Regional Medical Research Centre, Bhubaneswar, India

I have gone through the manuscript entitled *, **“*** ***Effect of hyperglycemia on lung microbiota and treatment outcome in pulmonary tuberculosis: A scoping review*** ***” *** carefully.

 It is a very good study and presented clearly.

**Comments :**

1. There are spelling mistakes at more than 4 places. Plz. correct these.
2. First of all, the authors need to adopt a uniform style of writing. The entire manuscript should be written in Times New Roman, Font 12 with double spacing. The present one is Times New Roman, Font 12 with 1.5 paragraph spacing.
3. **Abstract : **Include the place of study in Keywords. It is important.
4. The methodology, i.e., selection criteria of studies included, excluded, screened, assessed for eligibility, etc. in the review need to be represented in a figure.

If the authors can incorporate the suggested changes in the study, it would really improve the presentation.

Are the rationale for, and objectives of, the Systematic Review clearly stated?

Yes

Is the statistical analysis and its interpretation appropriate?

Yes

If this is a Living Systematic Review, is the ‘living’ method appropriate and is the search schedule clearly defined and justified? (‘Living Systematic Review’ or a variation of this term should be included in the title.)

Yes

Are sufficient details of the methods and analysis provided to allow replication by others?

Yes

Are the conclusions drawn adequately supported by the results presented in the review?

Yes

Reviewer Expertise:

NA

I confirm that I have read this submission and believe that I have an appropriate level of expertise to confirm that it is of an acceptable scientific standard, however I have significant reservations, as outlined above.

# Reviewer report: Reviewer response for version 1

**DOI:** 10.5256/f1000research.175303.r360302

**Published:** 2025-02-11

**Authors:** Foluke Adenike Ayeni​

**Affiliations:** 1 Department of Clinical Pharmacy and Biopharmacy, Faculty of Pharmacy, University of Lagos, Akoka, Lagos, Nigeria

This article is a scoping review that explores the effect of hyperglycemia on lung microbiota and treatment outcomes in patients with pulmonary tuberculosis (TB) and diabetes mellitus (DM). It revealed that hyperglycemia and lung microbiota are involved in the pathogenesis of DM-TB, which culminates in poor treatment outcomes in pulmonary TB.

 The objectives of the review are well-defined and relevant to the field of pulmonary tuberculosis (TB) and hyperglycemia. The study clearly aims to explore the relationship between hyperglycemia and lung microbiota, as well as its impact on TB treatment outcomes. The objectives address an important gap in the literature, and the approach is highly timely given the increasing prevalence of both TB and diabetes worldwide.

 The method used in this scoping review is thorough and appropriate for the topic at hand. The systematic search strategy is comprehensive, and the inclusion/exclusion criteria are well-justified. The reviewer’s approach to categorizing and summarizing the literature is rigorous, providing a clear framework for understanding the complexities of hyperglycemia's effect on lung microbiota and TB treatment outcomes. 

 The results are presented clearly and systematically. The synthesis of existing studies is balanced, providing a comprehensive overview of the current state of research while also identifying areas of uncertainty. The results will undoubtedly be valuable for future research and clinical applications.

 The discussion section is insightful and well-rounded. The authors effectively interpret the findings, connecting them to existing literature and providing context for their implications in TB treatment. They thoughtfully highlight the importance of understanding the interplay between hyperglycemia and the lung microbiota, particularly in diabetic TB patients. 

 Finally, the conclusions captured and supported the results obtained in the review.

Are the rationale for, and objectives of, the Systematic Review clearly stated?

Yes

Is the statistical analysis and its interpretation appropriate?

Not applicable

If this is a Living Systematic Review, is the ‘living’ method appropriate and is the search schedule clearly defined and justified? (‘Living Systematic Review’ or a variation of this term should be included in the title.)

Not applicable

Are sufficient details of the methods and analysis provided to allow replication by others?

Yes

Are the conclusions drawn adequately supported by the results presented in the review?

Yes

Reviewer Expertise:

TB-DM comorbidity.

I confirm that I have read this submission and believe that I have an appropriate level of expertise to confirm that it is of an acceptable scientific standard.
